# The correction of the diastasis of the rectus abdominis muscle concomitant with a moulded silicone implant insertion in a patient with medial pectus excavatum

**DOI:** 10.1093/icvts/ivac147

**Published:** 2022-05-30

**Authors:** Jaime Anger, Jose Ribas Milanez de Campos

**Affiliations:** Hospital Israelita Albert Einstein, Av. Albert Einstein, 627, Sao Paulo, SP 05652900, Brazil; Hospital Israelita Albert Einstein, Av. Albert Einstein, 627, Sao Paulo, SP 05652900, Brazil

**Keywords:** Pectus excavatum, Rectus adominis muscle, Silicone implants

## Abstract

The association of the diastasis of the rectus abdominis muscle and the medial pectus excavatum was reported. We have been using soft silicone block, sculpted intraoperatively, to correct pectus excavatum. The horizontal access used, 2 cm at a subxiphoid position, allows us to expose the sternum and the rectus abdominis muscles (RAMs). We report a case, male, 31 years presenting medial pectus excavatum and supraumbilical diastasis of the rectus abdominis muscle with a width of 35 mm at the costal arches, and 27 mm at 6 cm from the xiphoid process edge. The muscle borders presented a curved lateral deviation up to the insertion in the costal arches. The necessary space for the implant was dissected and the block was sculpted. The medial and superior aponeurosis borders of the RAM were incised at 6 cm from the xiphoid, and the posterior border of the RAM was released. The aponeurosis borders were brought together, promoting a medial and anterior positioning of the RAM. The inferior border of the implant was attached to the raw superior borders of the RAM. The result was considered satisfactory, and a magnetic resonance image 14 months after showed continuity of the implant and the muscles, promoting a uniform body contour.

Registry: CAAE63181616.7.0000.0071.

## INTRODUCTION

Pectus excavatum (PE) is a chest congenital deformity presenting a depression of the sternum and costal cartilages in the anterior chest wall. The most frequent alteration occurs in the medial region of the anterior thorax with maximum recess located at the junction of thorax and abdomen [1]. In mild deformities, we have used a solid silicone block, which is trimmed and moulded during surgery [2]. We report a case presenting medial PE and diastasis of the rectus abdominis muscle where we use a silicone implant with simultaneous diastasis of the rectus abdominis muscle correction to improve aesthetic results.

## PATIENTS AND METHODS

Male, 31 years old, Haller index 2.54. The bone defect dimensions were written down, and a soft solid silicone block was ordered (hardness 10 Shore A) and shaped as a parallelepiped. Surgical technique: a 7-cm incision access was made horizontally, 2 cm inferior to the xiphoid appendix. Dissection was performed on a supraperiosteal plane, in the sternum region, coinciding with the defect and done inferiorly to the access incision for 6 cm on the suprafascial plane, exposing the rectus abdominis muscles. The presence of diastasis of the rectus abdominis muscle was recognized. The distance was measured with a calliper (2.7 cm wide at 4 cm below the xiphoid, and 3.5 cm at the costal insertion).

After dissection, the block’s posterior wall was sculpted, creating a mirror image of the contour of the osteocartilaginous structures. Afterwards, the anterior face of the block was sculpted to mimic the contour of the sternum and ribs.

A vertical incision was done bilaterally at the medial edge of the rectus abdominis muscles (RAMs), 6 cm from the xiphoid appendix through the superior edge and continued laterally up to the costal insertion. The posterior and superior borders of the muscles were released from the posterior fascia and costal insertions. Then, the medial edges of the anterior fascia were brought together with separated stitches using unabsorbable thread 2-0, promoting a junction of the muscle edges medially in an anterior anatomically new position. The implant was inserted permanently, and its inferior edge was joined to the superior sutured RAM fascia with 2 non-absorbable sutures, assuming the same spatial position. The subcutaneous tissue and the skin were joined using 4-0 absorbable thread.

**Figure 1:**
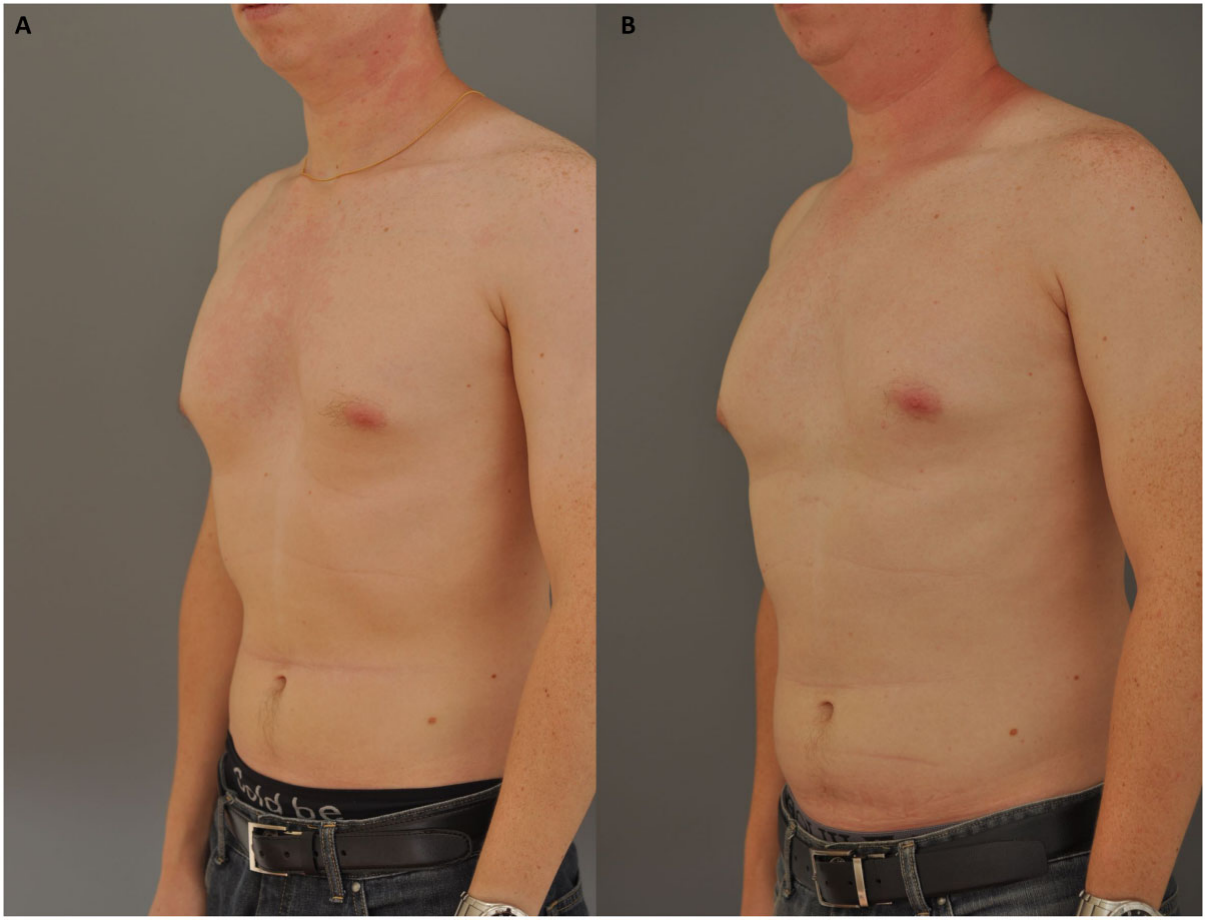
(**A**) Preoperative lateral view. (**B**) Six-year postoperative view.

**Figure 2:**
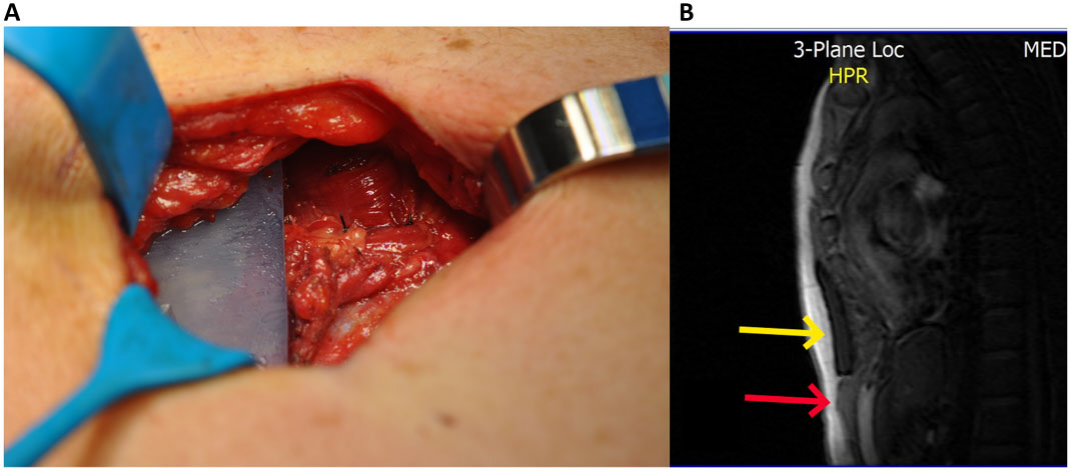
Intraoperative view. (**A**) The inferior edge of the implant is in direct contact with the superior sutured edges of the rectus abdominis muscles. (**B**) Magnetic resonance image: the superior arrow indicates the silicone implant and the inferior arrow indicates the rectus abdominis muscles.

The patient considered the result satisfactory (Fig. 1). Magnetic resonance imaging of the area was done 14 months after the surgery, showing the continuity of the anterior contour of the silicone block and the RAM, resembling the intraoperative final view (Fig. 2).

## DISCUSSION

Descriptions of the physical aspect of the medial PE focus on the depressed position of the sternum and the final costal arches and emphasize a ‘typical pectus posture’, illustrated by a forward-wrapping curvature, a relative prominence of the lower costal arches, and a configuration of the depressed abdomen in continuation with the PE, which becomes more prominent towards the hypogastrium [1]. The separation of RAMs in continuity with the sternum cavity associated with a patient’s posture may highlight the appearance of the depression even more [3]. The case we present may indicate the necessity to re-evaluate the muscular changes and surgical procedures, and possibly include the muscular surgical assessment to improve the aesthetic result. No similar surgical technique was reported before.

**Conflict of interest:** none declared.

## Reviewer information

Interactive CardioVascular and Thoracic Surgery thanks Guven Olgac, Bedrettin Yildizeli and the other anonymous reviewer(s) for their contribution to the peer review process of this article.
